# Supplementary material for: Mechanisms governing the pioneering and redistribution capabilities of the non-classical pioneer PU.1
Source: Nat Commun. 2020 Jan 21;11:402. doi: 10.1038/s41467-019-13960-2 (PMC6972792; doi:10.1038/s41467-019-13960-2)
Supplement: Supplementary file 7 — Source data [file 41467_2019_13960_MOESM7_ESM.zip › Source_Data/Figure6/Figure6B_MotifScanOutput/FLI1_mutPU.1/homerResults/motif51.similar.html]

motif51

## Information for motif51

T
C
A
G
C
A
G
T
A
C
G
T
G
T
A
C
C
G
A
T
A
G
T
C
A
C
T
G
A
G
T
C
A
C
T
G
T
C
G
A
  
Reverse Opposite:  

A
G
C
T
A
G
T
C
A
C
T
G
A
G
T
C
A
C
T
G
C
G
T
A
C
A
T
G
G
T
C
A
G
T
C
A
A
G
T
C
  

|  |  |
| --- | --- |
| p-value: | 1e-19 |
| log p-value: | -4.376e+01 |
| Information Content per bp: | 1.738 |
| Number of Target Sequences with motif | 86.0 |
| Percentage of Target Sequences with motif | 0.84% |
| Number of Background Sequences with motif | 74.0 |
| Percentage of Background Sequences with motif | 0.19% |
| Average Position of motif in Targets | 98.3 +/- 56.4bp |
| Average Position of motif in Background | 89.3 +/- 78.4bp |
| Strand Bias (log2 ratio + to - strand density) | 0.0 |
| Multiplicity (# of sites on avg that occur together) | 1.12 |
| Motif File: | file (matrix) reverse opposite |

### Similar de novo motifs found

|  |  |  |  |  |  |  |  |
| --- | --- | --- | --- | --- | --- | --- | --- |
| Rank | Match Score | Redundant Motif | P-value | log P-value | % of Targets | % of Background | Motif file |
| 1 | 0.930 | T C A G G C T A A G C T G A T C A G C T G T A C A C T G A G T C A C T G T G C A A C T G T C G A | 1e-18 | -41.512846 | 0.92% | 0.24% | motif file (matrix) |
| 2 | 0.878 | G T A C A C G T G T A C A C G T G T A C A C T G A T G C A C T G T G C A C A T G T C G A | 1e-15 | -35.230385 | 0.79% | 0.21% | motif file (matrix) |
| 3 | 0.949 | A C G T G T A C C G A T A G T C A C T G A G T C A C T G C G T A | 1e-15 | -34.706329 | 0.67% | 0.16% | motif file (matrix) |
| 4 | 0.888 | T C G A G T C A A G C T T G A C A G C T A G T C A C T G G A T C A C T G C G T A C A T G T C G A C A G T | 1e-14 | -32.801236 | 0.61% | 0.14% | motif file (matrix) |
| 5 | 0.887 | A C T G T G C A C T A G G T C A G A C T G A T C A G C T A G T C A C T G T A G C A C T G C G T A T C A G C T G A | 1e-13 | -31.477239 | 0.71% | 0.20% | motif file (matrix) |
